# Supplementary material for: Drug delivery systems for ovarian cancer treatment: a systematic review and meta-analysis of animal studies
Source: PeerJ. 2015 Dec 10;3:e1489. doi: 10.7717/peerj.1489 (PMC4690347; doi:10.7717/peerj.1489)
Supplement: Supplemental Information 1 [file peerj-03-1489-s001.docx]

# Supplemental material

## Search strategy

### PubMed

Search component 1:

**Drug delivery systems**

**Search component 1a:**

("Drug delivery systems"[MESH] OR "Liposomes"[MESH] OR "Micelles"[MESH] OR "Nanoparticles"[MESH] OR "Virosomes"[MESH] OR "Delayed-action preparations"[MESH] OR"Pharmaceutical Vehicles"[MESH]

OR

Doxil[tiab] OR Caelyx[tiab] OR Nanotax[tiab] OR Genexol-PM[tiab] OR Myocet[tiab] OR Xyotax[tiab] OR Paclical[tiab] OR Abraxane[tiab] OR Biocapsul*[tiab] OR Controlled-Release Prepar*[tiab] OR Delayed-action prepar*[tiab] OR Delivery system[tiab] OR Delivery systems[tiab] OR Dendrimer*[tiab] OR Dendrite[tiab] OR Dendritic Compound*[tiab] OR Dendritic Polymer*[tiab] OR Dendron*[tiab] OR Drug carrier*[tiab] OR Drug targeting*[tiab] OR Drugcarrier*[tiab] OR Hollow particle[tiab] OR Hollow particles[tiab] OR Liposom*[tiab] OR Micell*[tiab] OR Micro capsul*[tiab] OR Microcapsul*[tiab] OR Micro partic*[tiab] OR Micropartic*[tiab] OR Micro spher*[tiab] OR Microspher*[tiab] OR Nano capsul*[tiab] OR Nanocapsul*[tiab] OR Nano partic*[tiab] OR Nanopartic*[tiab] OR Niosom*[tiab] OR Pharmaceutical Vehicle*[tiab] OR Polymersom*[tiab] OR Prolonged-Action Prepar*[tiab] OR Sustained-Release Prepar*[tiab] OR Timed-Release Prepar*[tiab] OR Transferosom*[tiab] OR Virosom*[tiab] ] OR biocapsul*[tiab] OR nano biocapsule*[tiab] OR nanobiocapsul*[tiab] OR nano carrier[tiab] OR nano carriers[tiab] OR nanocarrier[tiab] OR nanocarriers[tiab] OR nano sphere*[tiab] OR nanosphere*[tiab] OR nano spheric*[tiab] OR nanospheric*[tiab] OR nano particl*[tiab] OR nanoparticl*[tiab] OR nano particulat*[tiab] OR nanoparticulat*[tiab] OR nano Vehicle[tiab] OR nano Vehicles[tiab] OR nanoVehicle[tiab] OR nanoVehicles[tiab] OR Nano Vesicle[tiab] OR Nano Vesicles[tiab] OR nanoVesicle[tiab] OR nanoVesicles[tiab] OR nano vesicular*[tiab] OR nanovesicular*[tiab] OR micro biocapsule*[tiab] OR microbiocapsul*[tiab] OR micro carrier[tiab] OR micro carriers[tiab] OR microcarrier[tiab] OR microcarriers[tiab] OR micro sphere*[tiab] OR microsphere*[tiab] OR micro spheric*[tiab] OR microspheric*[tiab] OR micro particl*[tiab] OR microparticl*[tiab] OR micro particulat*[tiab] OR microparticulat*[tiab] OR micro vesicular*[tiab] OR microvesicular*[tiab] OR micro Vehicle[tiab] OR micro Vehicles[tiab] OR microVehicle[tiab] OR microVehicles[tiab] OR Micro Vesicle[tiab] OR Micro Vesicles[tiab] OR microVesicle[tiab] OR microVesicles[tiab])

OR

**Search component 1b:**

(antineoplastic agents[MESH] OR Antineoplastic Agents[Pharmacological Action] OR Pharmaceutical Preparations[MESH] OR Cytostatic agents[MESH] OR chemotherap*[tiab] OR Cytostat*[tiab] OR Drug[tiab] OR Drugs[tiab] OR Medicin*[tiab] OR Pharmaceutical Preparat*[tiab] OR pharmaceutical agent[tiab])

AND

( capsul*[tiab] OR carrier[tiab] OR carriers[tiab] OR encapsul*[tiab] OR particl*[tiab] OR particulat*[tiab] OR sphere*[tiab] OR spheric*[tiab] OR Vehicle[tiab] OR Vehicles[tiab] OR Vesicle[tiab] OR Vesicles[tiab] OR Vesicular*[tiab] )

Search component 2:

**Ovarian cancer**

Ovarian Neoplasms [MESH] OR Meigs syndrome[tiab]OR Brenner tumor [tiab] OR Luteoma [tiab]

OR (Cancer[tiab] OR Cancers[tiab] OR Carcinoma*[tiab] OR Adenocarcinoma*[tiab] OR Adeno-carcin*[tiab] OR Cystadenocarcin*[tiab] OR Neoplas*[tiab] OR Dysplas*[tiab] OR Hyperplas*[tiab] OR Tumor[tiab] OR Tumors[tiab] OR Tumour*[tiab] OR Malignan* [tiab]) AND (Ovar*[tiab] OR Brenner [tiab] OR Adnex[tiab] OR Adnexa*[tiab] OR Fallopian tube*[tiab]) NOT ovariectomy[MESH]

### EMBASE

Search component 1:

**Drug delivery systems**

**Search component 1a:**

Exp drug delivery system/ OR Exp nanoparticle/ OR Exp controlled release formulation/ OR

(Doxil or Caelyx or Nanotax or Genexol-PM or Myocet or Xyotax or Paclical or Abraxane or Biocapsul* or Controlled-Release Prepar* or Delayed-action prepar* or Delivery system or Delivery systems or Dendrimer* or Dendrite or Dendritic Compound* or Dendritic Polymer* or Dendron* or Drug carrier* or Drug targeting* or Drugcarrier* or Hollow particle or Hollow particles or Liposom* or Micell* or Micro capsul* or Microcapsul* or Micro partic* or Micropartic* or Micro spher* or Microspher* or Nano capsul* or Nanocapsul* or Nano partic* or Nanopartic* or Niosom* or Pharmaceutical Vehicle* or Polymersom* or Prolonged-Action Prepar* or Sustained-Release Prepar* or Timed-Release Prepar* or Transferosom* or Virosom* or biocapsul* or nano biocapsule* or nanobiocapsul* or nano carrier or nano carriers or nanocarrier or nanocarriers or nano sphere* or nanosphere* or nano spheric* or nanospheric* or nano particl* or nanoparticl* or nano particulat* or nanoparticulat* or nano Vehicle or nano Vehicles or nanoVehicle or nanoVehicles or Nano Vesicle or Nano Vesicles or nanoVesicle or nanoVesicles or nano vesicular* or nanovesicular* or micro biocapsule* or microbiocapsul* or micro carrier or micro carriers or microcarrier or microcarriers or micro sphere* or microsphere* or micro spheric* or microspheric* or micro particl* or microparticl* or micro particulat* or microparticulat* or micro vesicular* or microvesicular* or micro Vehicle or micro Vehicles or microVehicle or microVehicles or Micro Vesicle or Micro Vesicles or microVesicle or microVesicles).ti,ab,kw.

OR

**Search component 1b:**

Exp pharmaceutics/ OR Exp antineoplastic agent/ OR ((chemotherap* or Cytostat* or Drug or Drugs or Medicin* or Pharmaceutical Preparat* or pharmaceutical agent).ti,ab,kw.)

AND

((capsul* or carrier or carriers or encapsul* or particl* or particulat* or sphere* or spheric* or Vehicle or Vehicles or Vesicle or Vesicles or Vesicular*).ti,ab,kw.)

Search component 2:

**Ovarian cancer**

exp ovary tumor/ OR (Meigs syndrome or Brenner tumor or Luteoma).ti,ab,kw. OR ((Cancer or Cancers or Carcinoma* or Adenocarcinoma* or Adeno-carcin* or Cystadenocarcin* or Neoplas* or Dysplas* or Hyperplas* or Tumor or Tumors or Tumour* or Malignan*).ti,ab,kw. )

AND

(Ovar* or Brenner or Adnex or Adnexa* or Fallopian tube*).ti,ab,kw.

## Risk of bias analysis

List of items:

1. (Reporting) is it mentioned that the experiment was randomized?
2. (Bias)Was the allocation sequence adequately generated and applied?
3. Were the groups similar at baseline or was adjusted for confounders in the analysis?
4. (Reporting) Is it mentioned that the experiment was blinded (level unknown)?
5. (Bias)Was the allocation adequately concealed?
6. (Bias) Are the animals randomly housed during the experiment?
7. (Bias) Were the caregivers/ and or investigators during the course of the experiment blinded from knowledge of which intervention each animal received?
8. (Bias) Were animals selected at random for the outcome assessment?
9. (Bias) Was the outcome assessor blinded?
10. (Bias) Were incomplete outcome data adequately addressed?
11. (Bias) Was the study apparently free of other problems that could pose a high risk of bias?
12. (Reporting) Is a power/sample size calculation shown?

## Risk of bias scores individual studies

|  | 1. Is it mentioned that the experiment was randomized? | 2. Is it mentioned that the experiment was blinded (level unknown)? | 3. Is a power/sample size calculation shown? | 4. Was the allocation sequence adequately generated and applied? | 5. Was the allocation adequately concealed? | | 6. Were the caregivers/ and or investigators during the course of the experiment blinded from knowledge of which intervention each animal received? | | 7. Was the outcome assessor blinded? | 8. Are the animals randomly housed during the experiment? | 9. Were animals selected at random for the outcome assessment? | 10. Were the groups similar at baseline or was adjusted for confounders in the analysis? | 11. Were incomplete outcome data adequately addressed? | 12. Was the study apparently free of other problems that could pose a high risk of bias? | Remarks: |
| --- | --- | --- | --- | --- | --- | --- | --- | --- | --- | --- | --- | --- | --- | --- | --- |
| Alagkiozidis *et al.* 2009 | n | n | n | ? | | ? | | ? | ? | ? | ? | ? | ? | ? |  |
| Ali *et al.* 2001 | n | n | n | ? | | ? | | ? | ? | ? | ? | ? | ? | ? |  |
| Ateh *et al.* 2011 | n | n | n | ? | | ? | | ? | ? | ? | ? | ? | ? | n | 1 |
| Chaudhury *et al.* 2012 | y | y | n | ? | | ? | | ? | y | ? | ? | ? | ? | y |  |
| Cho *et al.* 2013 | n | n | n | ? | | ? | | ? | ? | ? | ? | ? | ? | ? |  |
| Cho *et al.* 2014 | n | n | n | ? | | ? | | ? | ? | ? | ? | ? | ? | ? |  |
| Cirstoiu *et al.* 2010 | y | n | n | ? | | ? | | ? | ? | ? | ? | y | n | ? |  |
| Daoud *et al.* 1994 | n | n | n | ? | | ? | | ? | ? | ? | ? | ? | ? | ? |  |
| Gao *et al.* 2005 | y | n | n | ? | | ? | | ? | ? | ? | ? | ? | ? | ? | 2 |
| Gharpure *et al.* 2014 | n | n | n | ? | | ? | | ? | ? | ? | ? | ? | ? | n | 1 |
| Gilmore *et al.* 2012 | n | n | n | ? | | ? | | ? | ? | ? | ? | ? | ? | ? |  |
| Javid *et al.* 2014 | n | n | n | ? | | ? | | ? | ? | ? | ? | ? | ? | ? |  |
| Jin *et al.* 2007 | n | n | n | ? | | ? | | ? | ? | ? | ? | ? | ? | ? |  |
| Konishi *et al.* 2012 | y | n | n | ? | | ? | | ? | ? | ? | ? | ? | ? | ? |  |
| Lee *et al.* 2013 | y | n | n | ? | | ? | | n | ? | n | ? | ? | ? | ? |  |
| Li and Howell 2010 | n | n | n | ? | | ? | | ? | ? | ? | ? | ? | ? | ? |  |
| Lu *et al.* 2006 | y | n | n | ? | | ? | | ? | ? | ? | ? | ? | ? | ? |  |
| Lu *et al.* 2007 | y | ? | n | ? | | ? | | ? | ? | ? | ? | ? | ? | ? |  |
| Lu *et al.* 2008 | n | n | n | ? | | ? | | ? | ? | ? | ? | ? | ? | ? |  |
| Mantia-Smaldone *et al.* 2014 | n | n | n | ? | | ? | | ? | ? | ? | y | ? | ? | ? |  |
| Mignard *et al.* 2010 | y | n | n | ? | | ? | | ? | ? | ? | ? | y | ? | n | 1 |
| Paraskar *et al.* 2010 | n | n | n | ? | | ? | | ? | ? | ? | ? | n | ? | ? | 3 |
| Pastorino *et al.* 2008 | n | n | n | ? | | ? | | ? | ? | ? | ? | ? | ? | ? |  |
| Patankar *et al.* 2013 | n | n | n | ? | | ? | | ? | ? | ? | ? | n | ? | ? | 2, 3 |
| Patankar *et al.* 2013-1 | n | n | n | ? | | ? | | ? | ? | ? | ? | ? | ? | ? | 2 |
| Perkins *et al.* 2000 | n | n | n | ? | | ? | | ? | ? | ? | ? | ? | ? | ? |  |
| Pu *et al.* 2014 | y | y | n | ? | | ? | | ? | ? | ? | ? | ? | ? | ? |  |
| Rapoport *et al.* 2004 | y | n | n | ? | | ? | | ? | ? | ? | ? | ? | ? | ? |  |
| Sengupta *et al.* 2012 | n | n | n | ? | | ? | | ? | ? | ? | ? | n | ? | ? | 3 |
| Shaikh *et al.*2 013 | y | y | n | ? | | ? | | ? | ? | ? | ? | ? | ? | ? |  |
| Storm *et al.*. 1994 | n | n | n | ? | | ? | | ? | ? | ? | ? | ? | ? | ? | 4 |
| Tang *et al.* 2012 | n | n | n | ? | | ? | | ? | ? | ? | ? | ? | ? | ? |  |
| Tong *et al.* 2014 | y | n | n | ? | | ? | | ? | ? | ? | y | ? | ? | ? |  |
| Ueno *et al.* 1988 | n | n | n | ? | | ? | | ? | ? | ? | ? | y | ? | ? |  |
| Vaage *et al.* 1993 | n | n | n | ? | | ? | | ? | ? | ? | ? | ? | ? | ? |  |
| Vingerhoeds *et al.* 1996 | n | n | n | ? | | ? | | ? | ? | ? | ? | ? | ? | ? |  |
| Werner *et al.* 2011 | n | n | n | ? | | ? | | ? | ? | ? | ? | ? | ? | n | 1, 2 |
| Winer *et al.* 2010 | y | n | n | n | | ? | | ? | ? | ? | ? | ? | ? | ? | 2, 5 |
| Xiao *et al.* 2009 | y | n | n | ? | | ? | | ? | ? | ? | ? | y | ? | ? | 2 |
| Xu *et al.* 2006 | n | n | n | ? | | ? | | ? | ? | ? | ? | ? | ? | ? |  |
| Yang *et al.* 2014 | n | y | n | ? | | ? | | ? | ? | ? | ? | y | ? | ? |  |
| Ye *et al.* 2013 | y | n | n | ? | | ? | | ? | ? | ? | ? | ? | ? | ? |  |
| Zeng *et al.* 2013 | y | n | n | ? | | ? | | ? | ? | ? | ? | ? | ? | ? |  |
| Zhang *et al.* 2013 | y | n | n | ? | | ? | | ? | ? | ? | ? | ? | n | ? |  |
| Yes | 39% | 9% | 0% | 0% | | 0% | | 0% | 2% | 0% | 5% | 11% | 0% | 2% |  |
| Unknown / not described | 0% | 2% | 0% | 98% | | 100% | | 98% | 98% | 98% | 95% | 82% | 95% | 89% |  |
| No | 61% | 89% | 100% | 2% | | 0% | | 2% | 0% | 2% | 0% | 7% | 5% | 9% |  |

Remarks:

1. Potential conflict of interest
2. Not blinded while using a humane endpoint
3. Differences at baseline
4. No clear materials and methods
5. Incorrect randomization method

## Supplemental material to Figure 3. Experimental details on specific experiments in meta-analysis survival data:

1. Chaudhury et al. 2012: carboplatin loaded non targeted liposomes vs. free carboplatin (15 mg/kg; IP)
2. Yang et al.2014: paclitaxel-microspheres vs. free paclitaxel (20 mg/kg; IP)
3. Cirstoiu-Hapca et al. 2010: Treatment initiated on day 3: Nanoparticles-paclitaxel-rituximab (anti-CD20) vs. free paclitaxel (initial: 20 mg/kg; subsequent 10 mg/kg; IV/IP)
4. Cirstoiu-Hapca et al. 2010: Treatment initiated on day 3: Nanoparticles-paclitaxel-trastuzumab (anti-HER2) vs. free paclitaxel (initial: 20 mg/kg; subsequent 10 mg/kg; IV/IP)
5. Konishi et al. 2012: irinotecan liposomes vs. free irinotecan hydrochloride (45 mg/kg; IV)
6. Pu et al. 2014: Targeted paclitaxel loaded microbubbles+ultrasound vs. free paclitaxel +ultrasound (20 mg/kg; IP)
7. Winer et al.2010*: F3-peptide-cisplatin-nanoparticles vs. blank+free cisplatin (75 μg/kg; IP)
8. Cirstoiu-Hapca et al. 2010: Treatment initiated on day 5: Nanoparticles-paclitaxel- rituximab (anti-CD20) vs. free paclitaxel (initial: 20 mg/kg; subsequent 10 mg/kg; IV/IP)
9. Shaikh et al. 2013: Encapsulated doxorubicin and irinotecan vs. free doxorubicin and free irinotecan (10 µmol/kg; IV)
10. Pastorino et al. 2008: TVT-doxorubicin vs. free doxorubicin (5 mg/kg; IV)
11. Pastorino et al. 2008: Caelyx vs. free doxorubicin (5 mg/kg; IV)
12. Patankar et al. 2013: Topophore C vs. free topotecan (5 mg/kg; IV)
13. Pu et al. 2014: Targeted paclitaxel loaded microbubbles vs. free paclitaxel (20 mg/kg; IP)
14. Winer et al.2010*: F3-peptide-cisplatin-nanoparticles vs. blank+free cisplatin (150 μg/kg; IP/IV)
15. Tong et al.2014: Folate-paclitaxel-liposomes vs. free paclitaxel (20 mg/kg; IP)
16. Cirstoiu-Hapca et al. 2010: Treatment initiated on day 5: Nanoparticles-paclitaxel- trastuzumab (anti-HER2) vs. free paclitaxel (initial: 20 mg/kg; subsequent 10 mg/kg; IV/IP)
17. Tang et al.2012: cisplatin-microparticles vs. free cisplatin (2 mg/kg; IP)
18. Winer et al.2010*: F3-peptide-cisplatin-nanoparticles vs. blank+free cisplatin (75 μg/kg IV)
19. Lee et al. 2013*: Paclitaxel-NS (25 mg/kg; IP) vs. free paclitaxel (15 mg/kg; IV)
20. Xiao et al.2009: paclitaxel nanoparticles vs. free paclitaxel (20 mg/kg; IP)
21. Li and Howell 2010*: Hyplat (CD44-targeted nanoparticles; 5 mg/kg; IP) vs. free cisplatin (10 mg/kg; IP)
22. Tong et al.2014: paclitaxel-liposomes vs. free paclitaxel (20 mg/kg; IP)
23. Rapoport et al. 2004: P-105 doxorubicin vs. free doxorubicin (3 mg/kg; IP)
24. Lu et al. 2008: Single dose priming tumor penetrating microparticles paclitaxel vs. single dose free paclitaxel (40 mg/kg; IP)
25. Tong et al.2014: Folate-paclitaxel-liposomes vs. free paclitaxel (20 mg/kg; IV)
26. Javid et al. 2014: doxorubicin-magnenite nanoparticles vs. free doxorubicin (10 mg/kg; IP)
27. Javid et al. 2014: paclitaxel-magnenite nanoparticles vs. free paclitaxel (10 mg/kg; IP)
28. Chaudhury et al. 2012: carboplatin loaded folate-liposomes vs. free carboplatin (15 mg/kg; IP)
29. Pu et al. 2014: Nontargeted paclitaxel loaded microbubbles+ultrasound vs. free paclitaxel+ultrasound (20 mg/kg; IP)
30. Pu et al. 2014: Nontargeted paclitaxel loaded microbubbles vs. free paclitaxel (20 mg/kg; IP)

## Supplemental material to Figure 4. Experimental details on specific experiments in meta-analysis:

1. Javid et al. 2014: Doxorubicin-magnenite nanoparticles vs. free doxorubicin (10 mg/kg; IP); tumor volume (mm3)
2. Javid et al. 2014: Paclitaxel-magnenite nanoparticles vs. free paclitaxel (10 mg/kg; IP); tumor volume (mm3)
3. Lu et al. 2007: paclitaxel-nanoparticles vs. free paclitaxel (5 mg/kg; IP); tumor weight (g)
4. Ateh et al. 2011: Paclitaxel-microparticles CD95ligand targeted vs. free paclitaxel (20 mg/kg; IP); relative radiance
5. Ateh et al. 2011: Paclitaxel-microparticles non-targeted vs. free paclitaxel (20 mg/kg; IP); relative radiance
6. Patankar et al. 2013-1*: Topophore C (2.5 mg/kg; IV) vs. free topotecan (15 mg/kg; IV); % increase in bioluminescence at day 42
7. Zhang et al. 2013: Nanobins(As) vs. free AsO3 (4 mg/kg; IP); tumor weight (g)
8. Yang et al. 2014: Paclitaxel-microspheres vs. free paclitaxel (20 mg/kg; IP); bioluminescence
9. Cirstoiu-Hapca et al. 2010: Nanoparticles-paclitaxel-rituximab (anti-CD20) vs. free paclitaxel (20 mg/kg; IV/IP); % bioluminescence at day 70
10. Zhang et al. 2013: Nanobins(As) urokinase plasminogen activator targeted vs. free AsO3 (4 mg/kg; IP); tumor weight (g)
11. Sengupta et al. 2012: cisplatin-nanoparticles vs. free cisplatin (3 mg/kg; IV); fold change bioluminescence
12. Xu et al. 2006: Cisplatin pH responsive nanoparticles vs. free cisplatin (10 mg/kg; IP); tumor nodule number
13. Paraskar et al. 2010: cisplatin-nanoparticles vs. free cisplatin (3 mg/kg; IV); bioluminescence fold change
14. Ye et al. 2013: Liposu (paclitaxel-liposomes) vs. free paclitaxel (5 mg/kg; IP); tumor weight
15. Li and Howell 2010*: Hyplat (CD44-targeted nanoparticles; 5 mg/kg; IP) vs. free cisplatin (10 mg/kg; IP); bioluminescence
16. Gilmore et al. 2012: Paclitaxel-expansile nanoparticles vs. free paclitaxel (10 mg/kg; IP); tumor mass (g)
17. Xiao et al. 2009: paclitaxel-nanoparticles vs. free paclitaxel (20 mg/kg; IP); bioluminescence AU
18. Jin et al. 2007: Cisplatin-poly(ethylene glycol)-2k-50% nanogels vs. cisplatin (10 mg/kg; IP); tumor/cm
19. Konishi et al. 2012: irinotecan-liposomes vs. free irinotecan hydrochloride (45 mg/kg; IV); ascites volume at day 31
20. Xu et al. 2006: Cisplatin pH nonresponsive nanoparticles vs. free cisplatin (10 mg/kg; IP); tumor nodule number
21. Jin et al. 2007: Cisplatin-poly(ethylene glycol)-2k-25% nanogels vs. cisplatin (10 mg/kg; IP); tumor/cm

## **Supplementary table 1. Full study characteristics of included studies.** The following characteristics are included: type of drug delivery system, material of drug delivery system, general preparation procedure, mean particle size, zeta potential, cytostatic drug, drug concentration in particles, release characteristics, modifications, targeting method, *in vivo* experimental details (species/strain/genotype/ no animals per group/ sex age, weight, cell type, inoculation area), experimental groups, administration route, dose, regime, inoculation time before start, follow-up time, drop-outs, tumor size evaluation method, outcome measures and side-effect measures.

| Reference | Type of delivery system | Material Delivery system | General preparation procedure | Mean particle size | Zeta-potential | Cytostatic drug |
| --- | --- | --- | --- | --- | --- | --- |
| Alagkiozidis *et al.* 2009 [1] | Doxil (liposomes) | HSPC, PEG-DSPE, and cholesterol | ND | ND | ND | DOX |
| Ali *et al.* 2001 [2] | Liposomes | DSPC and DPPE-GA | Lipids and taxenes were dissolved in DSPC, DPPE-GA and taxenes (84:10:6) and evaporated under nitrogen stream. Films were dried under vacuum, hydrated in NaCl, vortexed, heated (58°C) and cooled (0°C). Formulations were extruded through nucleopore filters to a final pore size of 100 nm. | 90-140 nm | ND | PTX and prodrugs |
| Ateh *et al.* 2011 [3] | Microparticles | PLGA | Placebo- or PTX- microspheres were prepared by filtering (0.2 µm) PLGA in dichloromethane and emulsifying it through a microsieve membrane into an aqueous solution with an emulsifier. This was stirred to evaporate the solvent and concentrated by filtration and washing. | 1.6 µm (placebo) and 1.6 µm (PTX-loaded) | ND | PTX |
| Chaudhury *et al.* 2012 [4] | Liposomes | DPPC/DSPE-PEG(-FA) or DSPC/DSPE-PEG(-FA) | DPPC/DSPE-PEG, DSPC/DSPE-PEG (95:5 ratio), DPPC/DSPE-PEG/DSPE-PEG-FA or DSPC/DSPE-PEG/DSPE-PEG-FA (95:4.8:0,2 ratio) were dissolved in chlorofom and dried under nitrogen gas to make a lipid film. Next, it was placed under vacuum to remove residual solvent and hydrated with NaCl. Finally, the samples were extruded through 100 nm membranes. | 100-110 nm | ND | CPT |
| Cho *et al.* 2013 [5] | Micelles | PEG-b-PCL | PTX, CYP, GSP, and PEG-b-PCL were dissolved in acetone, followed by a rapid addition of pre-warmed 0.9% saline or PBS at 60 °C with mixing. Acetone was evaporated under reduced pressure at 60 °C. Insoluble drugs were removed by centrifugation, followed by filtration with 0.22 μm nylon syringe filters. | 80-90 nm | ND | PTX, CYP, GSP |
| Cho *et al.* 2014 [6] | Micelles | PEG-b-PCL | PTX, CYP, GSP, and PEG-b-PCL were dissolved in acetone, followed by a rapid addition of pre-warmed 0.9% saline at 60°C with mixing. Acetone was evaporated under reduced pressure at 60 °C. Insoluble drugs were removed by centrifugation, followed by filtration with 0.22 μm nylon syringe filters. | 83 nm for targeting micelles. 45 nm mPEG micelles | ND | PTX, CYP, GSP |
| Cirstoiu-Hapca *et al.* 2010 [7] | Nanoparticles | PDLA | Taxane dissolved in acetone was added to a solution of PLA in acetone (drug to polymer ratio 1:10). The organic phase was mixed under stirring with aqueous phase containing poly(vinylalcohol) and magnesium chloride hexahydrate. Then, pure water was added to the NP suspension, and it was stirred. NPs were recovered by centrifugation, washed with pure water and lyophilized in the presence of trehalose before storage at 4 °C. | 237 nm | ND | PTX |
| Daoud 1994 [8] | Liposomes | DMPC, CHOL and PS | ND; DMPC, CHOL and PS in 10:4:1 molar ratio | ND | ND | VAL |
| Gao *et al.* 2005 [9] | Micelles | PEO-PPO-PEO and PEG-DSPE | Micelles were formed by mixing equal volumes of Pluronic P-105 and PEG2000-DSPE. | Pluronic P-105: 17.5 nm. Mixed micelles Pluronic P-105 and PEG2000-DSPE): 8.9 nm. | ND | DOX |
| Gharpure *et al.* 2014 [10] | Nanoparticles | PLGA | PLGA and docetaxel were spread on a sheet of poly(ethylene terephthalate) (PEt) with a Mayer Rod. Solvent was evaporated with heat. Sheet was placed in contact with mold and subsequently separated using a hot laminator at 130°C and 80 psi. The mold was then placed in contact with another PEt sheet coated with polyvinyl alcohol. Nanoparticles were then transferred from the mold to PET using a hot laminator and removed from the PET sheet by passing though rollers and applying water to dissolve the polyvinyl alcohol. | 227.7nm | ND | DOC |
| Gilmore *et al.* 2012 [11] | Nanoparticles | Methacrylate polymer | PTX dissolved in methacrylate monomer solution was mini-emulsified polymerized and dialyzed against phosphate buffer to remove excess surfactant and salts. | 100 nm | ND | PTX |
| Javid *et al.* 2014 [12] | Superparamagnetic iron oxide nanoparticles | PEG–APTES-SPIO | FeCl_3_.6H_2_O and FeCl_2_.4H_2_O (2:1) was stirred at 30°C (pH 6.9) for 45 min. Then, aqueous ammonia solution was added dropwise, under the cover of N_2_ gas, and the pH of the solution was carefully adjusted up to 10. Particles were then filtered and rinsed with deionized water, methanol, and toluene five times and dispersed in toluene at pH 7.0. | DOX: 27 nm and PTX 30 nm | ND | DOX or PTX |
| Jin *et al.* 2007 [13] | Nanogels | PAE and PEG | PAE-graft-PEG and CIS were dissolved in water (1:3:7 ratio) and stirred at RT or 70°C for 10 min and then at RT for 12 h. | 25% PEG-2k: 126 nm; 50% PEG-2k: 214 nm | P2K25-3: -23.7 mV; P2K50-3: -7.74 mV | CIS |
| Konishi *et al.* 2012 [14] | Liposomes | ND | ND | ND | ND | ITC |
| Lee *et al.* 2013 [15] | Nanosuspensions | DOTAP | PTX dissolved in N-methylpyrrolidone was precipitated in an aqueous solution containing one or more surfactants and other excipients. Crude suspension was then high-pressure homogenized to produce nanosuspensions, which were stabilized by surfactant coating added during centrifugation or re-homogenization steps. Organic solvents were removed by centrifugation. | 160-170 nm | 12.9 mV | PTX |
| Li and Howell 2010 [16] | Hyplat | Hyaluronan | Hyplat was produced by incubating a solution of CIS hyaluronan solution in deionized water at 90-95°C for 1 h followed by cooling on ice and dialysis against sterile water to remove unbound platinum. | 580.6 nm | 40.3 mV | CIS |
| Lu *et al.* 2006 [17] | Nanoparticles | PLA | PTX and PLA were dissolved in dichloromethane and an aqueous emulsifier was added. Solution was sheared at high-speed, and (ultrasonically) emulsified. Water was added and mixed under constant temperature in a water bath to evaporate the organic dissolvent. | About 200 nm | ND | PTX |
| Lu *et al.* 2007 [18] | Nanoparticles | PLA | PTX and PLA were dissolved in dichloromethane and an aqueous emulsifier was added. Solution was sheared at high-speed, and (ultrasonically) emulsified. Water was added and mixed under constant temperature in a water bath to evaporate the organic dissolvent. | About 200 nm | ND | PTX |
| Lu *et al.* 2008 [19] | Tumor-penetrating microparticles | PLG | PLG and PTX were co-dissolved in methylene chloride and emulsified in 1% PVA aqueous solution by homogenization for 30 s. The emulsion was mixed with 0.1% PVA and stirred at 1000 rpm at room temperature, and ambient pressure was used to evaporate the methylene chloride. The residual microparticles pellet was collected by centrifugation, washed three times with deionized water to remove residual PVA, lyophilized, and stored at 4°C. | Priming TPM: number-based diameter 3.6 µm and volume based diameter 5.7 µm. Sustaining TPM: number-based diameter: 3.8 µm and volume based diameter 5.2 µm | ND | PTX |
| Mantia-Smaldone *et al.* 2014 [20] | Doxoves (Liposomes) | HSPC/CHOL/mPEG-DSPE (from doxoves manufacturer) | ND | 85 nm (from doxoves manufacturer) | ND | DOX |
| Mignard *et al.* 2010 [21] | Micelles | DEBIO 0507 (DACH-platinum derivative) | ND | ND | ND | DACH-platinum derivative (oxaliplatin) |
| Paraskar *et al.* 2010 [22] | Nanoparticles | PIMA-GA | Poly(isobutylene-alt-maleic anhydride) was dissolved in dry dimethylformamide. Solvent and low molecular weight impurities were removed under vacuum and using dialysis. The solution was lyophilized to get PIMA. Poly(isobutylene-alt-maleic anhydride was dissolved in DMF to which diaza(1,3)bicycle(5.4.0]undecane and glucosamine were added, stirred at RT for 48 h and quenched by double destilled water. Organic solvent was evaporated under vacuum. NPs were engineered by dissolving the polymer in water containing CIS for 48 h. | 80 - 150 nm | ND | CIS |
| Pastorino *et al.* 2008 [23] | Liposomes | ND | ND | ND | ND | DOX |
| Patankar *et al.* 2013 [24] | Liposomes | DSPC and CHOL | DSPC and CHOL were dissolved in chloroform and then mixed to a final molar ratio of 55:45. This solution was then dried to a thin film under a gentle stream of nitrogen gas. The residual chloroform was removed under high vacuum. Dried lipid films were hydrated at 65°C by mixing with CuSO_4_. Following hydration, the sample was subjected to five freeze (liquid nitrogen) and thaw (65°C) cycles. The multilamellar vesicles were extruded 10 times through stacked polycarbonate filters of 0.1 μm and 0.08 μm pore size at 65°C. | 95-110 nm | ND | TOP |
| Patankar *et al.* 2013-1 [25] | Liposomes | DSPC and CHOL | See Patankar et al. 2013 [24]. | ND | ND | TOP and DOX |
| Perkins *et al.* 2000 [26] | Lipocores | BrC16-T and DSPE-PEG | Lipid and drug co-dissolved in ethanol. Solution was then injected into Hepes buffered saline, and heated to 60°C. This was vortexed to the final concentration of 10 mg/ml drug. | 50-100 nm | ND | PTX with acyl chain |
| Pu *et al.* 2014 [27] | Microbubbles | DSPC and DSPE | DSPC, DSPE and PTX were dissolved in 100% glycerin in PBS. This was incubated in 40°C water for 30 min, degassed, and reperfused with perfluoropropane gas. The mixture was then mechanically vibrated for 45 s in a dental amalgamator at a vibration frequency of 60 Hz. LHRHa was biotinylated and conjugated to avidinylated microbubbles. Free ligands were removed through washing with PBS. | Targeted PTX loaded microbubbles (TPLMBs):1.8 µm; nontargeted PTX loaded microbubbles (NPLMBs): 1.4 µm | TPLMBs: -9.6 mV; NPLMBs -8.5 mV | PTX |
| Rapoport *et al.* 2004 [28] | Micelles | PEO-PPO-PEO | ND | ND | ND | DOX |
| Sengupta *et al.* 2012 [29] | Nanoparticles | CHOL and succinic acid | PC, CHOL-CIS conjugate, and DSPE-PEG were dissolved in DCM. Solvent was evaporated into a thin and uniform lipid-drug film using a rotary evaporator. The lipid-drug film was then hydrated with H_2_O for 1 h at 60 °C. | 141.4 nm | ND | CIS |
| Shaikh *et al.* 2013 [30] | Liposomes | DSPC and CHOL | Lipids were dissolved in chloroform and evaporated under a stream of nitrogen gas to remove chloroform. Samples placed under vacuum for 3 h to remove solvent. Dried films were hydrated at 65 °C for 1 h using MnSO_4_ or MnCl_2_ (pH 3.5). Resultant multilamellar vesicles were extruded 10 times at 65 °C through stacked polycarbonate filters with pore sizes of 0.1 and 0.08 μm. | 100-110 nm | ND | DOX & ITC |
| Storm *et al.* 1994 [31] | Liposomes | MPB-PE | Extrusion of multilamellar liposomes through 0.6 and 0.2 µm polycarbonate membranes. DOX was encapsulated via aqueous hydration medium. | 250 nm | ND | DOX |
| Tang *et al.* 2012 [32] | Microparticles | Apoptopic cells | 2·10^7^ A2780 cells were treated with 300 ug/ml CIS and exposed to UVb radiation for 1 h. After 12 h, supernatant was centrifuged to remove cells and debris. Finally it was centrifuged again to pellet microparticles. | 100-1000 nm | ND | CIS |
| Tong *et al.* 2014 [33] | Liposomes | DSPE-PEG and FA | DSPE-PEG2000 or DSPE-PEG2000-FA was dissolved in methanol and chloroform (1:9, volume ratio), followed by addition of distilled water. Methanol and chloroform were then removed by ultrasonic emulsification, resulting in transparent micelle solutions. These were coupled to nano-paclitaxel by coincubation (drug-to-lipid ratio 50:1) by mixing and incubating at 60°C for 1 h resulting in a PEGylated nanopaclitaxel liposomes. | FA targeted: 140.5 nm. Nontargeted: 121.6 nm | ND | PTX |
| Ueno *et al.* 1988 [34] | Liposomes | Egg-PC and CHOL with sulfatide, PS or DP | Lipids were dissolved in chloroform, which was eliminated under reduced pressure with a rotary evaporator creating a homogeneous lipid film. Adriamycin (DOX) dissolved in saline was added, gently agitated under argon gas to let the lipid film swell. This was used as lipid suspension liquid and processed using ultrasound irradiation. Liposomes were filtered (0.8 µm) and passed through a column with sepharose Cl-2b in saline, to remove free DOX and separate liposome fractions. It was concentrated by Centriflo CF25 and sterilized using a 0.22 µm filter. | 30 nm | ND | DOX |
| Vaage *et al.* 1993 [35] | Doxil (liposomes) | CHOL, HSPC and DSPE with methoxy-PEG-DSPE | ND | 96 nm | ND | DOX |
| Vingerhoeds *et al.* 1996 [36] | Liposomes | Egg-PC, EPG, CHOL and MPB-PE | Mixture Egg-PC:EPG:CHOL:MPB-PE (molar ratio 38.1:4:32:1.9) in chloroform was evaporated. Film was flushed with nitrogen and hydrated in ammonium sulfate containing desferal. After 10 freeze-thaw cycles (-196°C to 60°C ) the resulting dispersion was filtered (0.6 and 0.2 µm) under nitrogen pressure. Fab’ fragments for targeting were covalently coupled to MPB-PE. | About 250 nm | ND | DOX |
| Werner *et al.* 2011 [37] | Nanoparticles | PLGA-lecithin-PEG | PLGA was dissolved in acetonitrile and added dropwise in heated aqueous solution under gentle stirring followed by 3 min of vortexing. Nanoparticles were self-assembled for 2 h with stirring under vacuum. To create FA targeted nanoparticles, (lecithin+DMPE-DTPA)/(DSPE-PEG + DSPE-PEG-Folate) (7:3 molar ratio) with a weight ratio of 15% to PLGA dissolved in 4% ethanol and heated to 65° C. | 75 nm | -35 mV | PTX |
| Winer *et al.* 2010 [38] | Nanoparticles | Poly-acrylamide | Hexane (45 ml) was stirred under a constant purge of argon. AOT and Brij were added with mixing under argon for 20 min. Acrylamide and APMA dissolved in PBS were mixed with AHM and sonicated for 5 min. This was then added to the hexane mixture and stirred for 20 min under argon protection. The polymerization reaction was started with fresh ammonium persulfate and TEMED and mixed for 12 h. Hexane was removed by evaporation and nanoparticles were precipitated with ethanol. Surfactant and unreacted monomers were removed by ethanol and water washings. | 24.4 nm | ND | CIS |
| Xiao *et al.* 2009 [39] | Micelles | Cholic acid and PEG | PTX and polymers were dissolved in chloroform, evaporated and a dry polymer-drug film was obtained. This was reconstituted in PBS followed by sonication for 2 h allowing to self-assemble PTX loaded micellar nanoparticles. These were filtered through 0.22 µm filters. | 56 nm | -1.62 mV to 1.46 mV | PTX |
| Xu *et al.* 2006 [40] | Micelles | PDEA-b-PEG, PCL-PDMA and PCL-b-PEG | Copolymer was dissolved in acetone and CIS was added. This solution was added dropwise in PBS and stirred overnight to form micelles. Acetone was removed under reduced pressure for 8 h. | PDEA-PEG: 81.9 nm; PCL-PEG: 79.75 nm; PCL-PDMA: 121.7 nm | PDEA-PEG: 9.09 mV; PCL-PEG: -14.6 mV; PCL-PDMA: 20.86 mV | CIS |
| Yang *et al.* 2014 [41] | Microspheres | PEG-PSA | PTX and PEG-PSA were dissolved in dichloromethane and emulsified into 1% w/w polyvinyl alcohol aqueous solution. Particles were hardened by allowing dichloromethane to evaporate at room temperature while stirring for 2 h, and then collected via centrifugation and washed twice in ultrapure water. | 14.2 µm | ND | PTX |
| Ye *et al.* 2013 [42] | Liposomes | CHOL and lecithin | PTX, lecithin and CHOL were dissolved in ethanol and stirred for 5 min. The organic solvent was evaporated under reduced pressure by a rotary evaporator at 50°C. The resulting membrane was dissolved in mannitol with lysine. This was filtered through a 0.22 µm filter and lyophilized. | About 400 nm | ND | PTX |
| Zeng *et al.* 2013 [43] | Cucumber mosaic virus cages | Cucumber mosaic virus | Cucumber mosaic virus (CMV) particles from CMV strain Fny infected cucumbers were purified from cucumber leaves. Viral RNA was disrupted with RNase treatment and removed by dialysis. | 29 nm | ND | DOX |
| Zhang *et al.* 2013 [44] | Nanobins | CHOL, DSPC, and DSPE-PEG | Mixture of CHOL, DSPC, and DSPE-PEG2000 (45:51:4 mol%) was dissolved in chloroform and evaporated to a thin film at 60°C under vacuum for 24 h. Dry lipid films were hydrated at 60°C for 1 h and subjected to 10 freeze-thaw cycles. The hydrated lipids were downsized to 100 nm with a Lipex extruder at 55°C using 200 nm and 100 nm polycarbonate membranes. | Nontargeted nanobins: 94.4 nm; Targeted nanobins: 104.4 nm | Nontargeted nanobins: -2.14 mV; Targeted nanobins: -3 mV | AT |

| Reference | Drug concentration in particles | Release characteristics | Modification/target/ligand (active/passive) | Species/strain/genotype/ no. per group/sex | Age | Weight | Cell type (no. of cells) | Inoculation area | Administration route |
| --- | --- | --- | --- | --- | --- | --- | --- | --- | --- |
| Alagkiozidis *et al.* 2009 | ND | ND | PEG (passive) | Mouse/C57BL6/-/5/F | 8 weeks | ND | ID8-Vegf (5·10^6^) | IP | IP |
| Ali *et al.* 2001  **Table 1 (continued)** | ND | ND | na (passive) | Mouse/CB17/SCID/5/F | ND | ND | OVCAR-3 (1·10^7^) | IP | IP |
| Ateh *et al.* 2011 | 25% w/w | ND | na/CD95ligand/CD95 (active) | Mouse/Balbc/Foxn1^nu^/5/F | ND | ND | IGROV-1-luc (5·10^6^) | IP | IP |
| Chaudhury *et al.* 2012 | Encapsulation efficiencies for DPPC-NT, DPPC-FRT, DSPC-NT, and DSPC-FRT liposomes: 25%, 23%, 16%, and 14%, respectively | 60-70% release in PBS after 72 h | PEG/FRa/folate (active) | Mouse/CB17/SCID/6/F | 6-8 weeks | ND | IGROV-1 (5·10^6^) | IP | IP |
| Cho *et al.* 2013 | Loading efficiency: PTX: 2%, CYP and GSP: 1% | Plateau at 60, 93 and 67% release for PTX, CYP and GSP in 72 h. | PEG (passive) | Mouse/-/Foxn1^nu^/4/F | 6-8 weeks | ND | ES-2-luc (1·10^6^) and SKOV3-luc (2·10^6^) | IP | IP |
| Cho *et al.* 2014 | ND for PTX, CYP and GSP. Loading efficiency 2% for DiR (fluorescent probe) | ND | PEG/phosphatidyl serine/GFNFRLKAGAKIRFGS (active) | Mouse/-/Foxn1^nu^/4/F | 6-8 weeks | ND | ES-2-luc (1·10^6^) | IP | IP/IV |
| Cirstoiu-Hapca *et al.* 2010 | Encapsulation efficiency: 78%  Drug loading: 7.8% | ND | na/anti-HER2 or anti-CD20/Trastuzumab or Rituximab (active) | Mouse/CB17/SCID/3/F | 5-7 weeks | 18-20 gram | SKOV-3 and SKOV-3-luc-D3 (5·10^6^) | IP | IP/IV |
| Daoud 1994 | ND | ND | na (passive) | Mouse/-/Foxn1^nu^/8-10/F | ND | ND | OVCAR-3 (1·10^7^ & 5·10^7^) | IP | IP |
| Gao *et al.* 2005 | Encapsulation efficiency: Pluronic P-105: 66.7%; mixed micelles: 91.5% | ND | Sonification of tissue/PEG (passive) | Mouse/-/Foxn1^nu^/≥10/F | 4 weeks | 23-26 gram | A2780 (2-3.5·10^6^) | IP | IP |
| Gharpure *et al.* 2014 | Drug loading: 17.1% | Burst release: 100% within 24 h | na (passive) | Mouse/-/athymic/10/F | ND | ND | HeyA8-luc (3·10^5^) or Skov3ip1 (1·10^6^) | IP and intraovarian | IP |
| Gilmore *et al.* 2012 | Encapsulation efficiency: 85% | After 24 h minimal release at pH7.4, 100% release at pH 5. | Swelling at pH 5 (passive) | Mouse/Nu/J/6-11/F | ND | 22-26 gram | OVCAR-3/-luc (1·10^6^) | IP | IP |
| Javid *et al.* 2014 | Dox: 70%; PTX: 61.5% | 90 and 93% for DOX and PTX at pH 6.0 after 24 h | Magnetic, PEG (passive) | Mouse/Balbc/immune deficient/12/F | 4-6 weeks | ND | A2780 and OVCAR-3 (1·10^7^) | IP | IP |
| Jin *et al.* 2007 | Drug loading efficiency: P2K25-3: 87.2%; P2K50-3: 87.0%  Pt content: P2K25-3: 5.7 wt%; P2K50-3: 2.2 wt% | 15% burst release followed by limited additional release up to 72 h | PEG (passive) | Mouse/-/Foxn1^nu^/4/F&M | 9-13 weeks | ND | SKOV3 (1·10^7^) | IP | IP |
| Konishi *et al.* 2012 | ND | ND | PEG (passive) | Mouse/Balbc/Foxn1^nu^/10/F | 4 weeks | ND | ES-2 (2·10^5^) | IP | IV |
| Lee *et al.* 2013 | 1% w/v | ND | DOTAP coating (passive) | Mouse/NCr/Foxn1^nu^/10/F | 5-6 weeks | Assumed to be 25 g | OVCAR-3-rfp (5·10^6^) | IP | PTX-nanosuspension (NS): IP and free PTX: IV |
| Li and Howell 2010 | Encapsulation efficiency: 49.0% | In 5% dextrose: up to 2 days no progressive release.  In 0.9% NaCl: 30% within 30 min, slow-sustained release for next 24 h.  In 9% NaCl: 55% within 30 min, slow-sustained release for next 24 h. | na/CD44/hyaluronan (active) | Mouse/Balbc/Foxn1^nu^/7-10/F | 5-6 weeks | ±20 gram | A2780-DsRed (5·10^6^) | IP | IP |
| Lu *et al.* 2006 | 70% encapsulation efficiency | ND | na (passive) | Rat/F344/-/5-15/F | 6-8 weeks | ND | NuTu19 (3·10^6^) | IP | IP |
| Lu *et al.* 2007 | 70% encapsulation efficiency | ND | na (passive) | Rat/F344/-/5-15/F | 6-8 weeks | ND | NuTu19 (3·10^6^) | IP | IP |
| Lu *et al.* 2008 | Loading efficiency: 4.1% | Priming TPM: 70% drug load release in 24 h.  Sustaining TPM: 1% release daily | Fast & slow releasing/na (passive) | Mouse/Balbc/Foxn1^nu^/8-17/F | ND | ND | SKOV-3 (2·10^7^) | IP | IP |
| Mantia-Smaldone *et al.* 2014 | ND | ND | PEG (passive) | Mouse/FVB/na/5-10/na | 6-8 weeks | ND | K5-TVA modified (5·10^6^) | IP | IP |
| Mignard *et al.* 2010 | ND | ND | na (passive) | Mouse/na/nu/na/na | ND | ND | OVCAR-3 (na) | IP | IP |
| Paraskar *et al.* 2010 | 175 µg CIS/mg polymer | pH 5.5: about 50% after 70 h.  pH 8.5: about 20% after 70 h. | pH dependent release (passive) | Mouse/-/K-rasLSL/+PtenFL/FL/3/na | na | na | Adenovirus-Cre recombinase | Intrabursal | IV |
| Pastorino *et al.* 2008 | ND | ND | PEG / CD13/asparagine-glycine-arginine (active) | Mouse/-/Foxn1^nu^/10-12/F | 5 weeks | ND | OVCAR-3 (2.5·10^5^) | IP | IV |
| Patankar *et al.* 2013 | Drug loading: 80% after 5 min, >98% after 60 min | About 50% after 24 h | na (passive) | Mouse/NCr/Fox1nu/≥8/F | ND | ND | ES-2 (1·10^5^) | IP | IV |
| Patankar *et al.* 2013-1 | ND | ND | na (passive) | Mouse/NCr/Fox1nu/6/F | ND | SKOV-3 model: 20-25 gram | ES-2 or SKOV3-luc-D3 (1·10^5^ & 5·10^6^) | IP | IV |
| Perkins *et al.* 2000 | ND | ND | PEG (passive) | Mouse/CB17/SCID/6-10/F | 6-7 weeks | ND | OVCAR3 (5·10^6^) | IP | IP or IV |
| Pu *et al.* 2014 | Entrapment efficiency: TPLMBs: 73.1%; NPLMBs: 96.5% | ND | Ultrasound applied/LHRH-r/LHRHa (active) | Mouse/Balbc/nu/7/F | 4-5 weeks | ND | A2780/DDP (8·10^6^) | IP | IP |
| Rapoport *et al.* 2004 | ND | ND | Sonication (passive) | Mouse/-/Foxn1^nu^/5/na | 3 weeks | ND | A2780 (1·10^6^) | IP | IP |
| Sengupta *et al.* 2012 | ND | pH dependent. ±5·10^6^ ng at pH 5.5; ±3·10^6^ ng at pH 7 | pH dependent release (passive) | Mouse/ K-rasLSL/+/PtenFL/FL/>3/na | ND | ND | Adenovirus-Cre recombinase | Intrabursal | IV |
| Shaikh *et al.* 2013 | Loading efficiencies >80%  Encapsulation efficiencies >90% | About 25 to 60% (depending on conditions) retained in liposomes after 72 h incubation | Co-delivery (passive) | Mouse/CB17/SCID/ND/ND | ND | ND | IGROV-1 (5·10^5^) | IP | IV |
| Storm *et al.* 1994 | ND | ND | na/OA3/OV-TL3 (active) | Mouse/NMRI/Foxn1^nu^/9-10/F | ND | ND | OVCAR-3 (2·10^7^) | IP | IP |
| Tang *et al.* 2012 | ND | ND | na (passive) | Mouse/Balbc/c-scid/12/F | 6-8 weeks | ND | A2780 (5·10^6^) | IP | IP |
| Tong *et al.* 2014 | Encapsulation efficiency: FA-NP 97%; nontargeted NP 99%. | ND | PEG, FRa/FA (active) | Mouse/Balbc/athymic/10/F | 4-6 weeks | ND | SKOV-3 (3·10^7^) | IP | IP or IV |
| Ueno *et al.* 1988 | Entrapped ADM/lipid ratio 19·10^3^ | ND | na (passive) | Mouse/Balbc/Foxn1^nu^/na/M | ND | 20 g | AMOC-1 (na) | ND | IV |
| Vaage *et al.* 1993 | Encapsulation efficiency >90% | ND | PEG (passive) | Mouse/Swiss/nu/nu/10/F | ND | ND | HEY (1·10^5^) | IP | IP |
| Vingerhoeds *et al.* 1996 | ND | ND | na/OA3/OV-TL3 (active) | Mouse/NMRI/Foxn1^nu^/3-4/na | 5-10 weeks | ND | OVCAR-3 (4·10^7^) | IP | IP |
| Werner *et al.* 2011 | ND | >90% after 24 h | PEG/FRa/FA (active) | Mouse/-/Foxn1^nu^/5-7/F | ND | ND | SKOV-3 (14·10^6^) | IP | IP |
| Winer *et al.* 2010 | Loading efficiency: 0.75 µg/mg nanoparticle | ND | na/CD13/F3 peptide (active) | Mouse/-/-/10/na | ND | ND | ID-8 (2·10^6^) | IP | IP or IV |
| Xiao *et al.* 2009 | Loading efficiency: almost 100% when initial amount of PTX was <25wt%, 82% and 73% when initial amount increased to 33 and 50wt%, respectively | 20% in first 2 h and a cumulative release of 35% by 12 h and 75% by 156 h in PBS | PEG (passive) | Mouse/- /Foxn1^nu^/5-6/F | 6-8 weeks | ND | SKOV3-luc (1·10^7^) | IP | IP |
| Xu *et al.* 2006 | Loading efficiency: PDEA-PEG: 97.8%; PCL-PEG: 73.9%; PCL-PDMA: 90.1%  Drug loading:  PDEA-PEG: 1.96wt%; PCL-PEG: 1.48wt%; PCL-PDMA: 1.8wt% | ND | pH-sensitive, PEG (passive) | Mouse/Balbc/Foxn1^nu^/4/ND | 6-8 weeks | ND | SKOV-3 (1·10^7^) | IP | IP |
| Yang *et al.* 2014 | Encapsulation efficiency: 67%. Drug loading: 13% | Almost 100% in 15 days | PEG (passive) | Mouse/C57BL6/-/5/F | 6-8 weeks | ND | MOSEC-luc (5·10^5^) | IP | IP |
| Ye *et al.* 2013 | Encapsulation efficiency: about 99% | ND | na (passive) | Rat/F344/-/6-21/F | 6-8 weeks | ND | NuTu19 (3·10^6^) | IP | IP |
| Zeng *et al.* 2013 | Encapsulation efficiency: ±60%.  Drug loading efficiency: 14.1% | ±80% after 120 h | na/FRa/FA (active) | Mouse/Balbc/Foxn1^nu^/6/ND | ND | ND | OVCAR-3 (5·10^6^) | IP | IP |
| Zhang *et al.* 2013 | ND | ND | PEG/uPA/ATN-291 (active) | Mouse/ - / athymic/5/F | 5-6 weeks | ND | Hey8-GFP (5·10^5^) | IP | IP |

| Reference | Dose | Regime | Inoculation time before treatment start | Follow-up time | Drop-outs | Tumor size evaluation method | Outcome measures | Side effect measures |
| --- | --- | --- | --- | --- | --- | --- | --- | --- |
| Alagkiozidis *et al.* 2009 | 2.5, 5, and 7.5 mg/kg | Weekly for 4 weeks | 1 week | ND | ND | na | Survival | ND |
| Ali *et al.* 2001  **Table 1 (continued)** | 50 mg/kg for taxane prodrugs. 12.5 mg/kg for taxol | Treatment on day 1, 3, 6, 7, 9 | 1 day | 300 days | Observations and mortalities recorded twice per week | na | Survival | ND |
| Ateh *et al.* 2011 | 20 mg/kg | Treatment once per week (day 7, 14, 21, 28) | 1 week | 62 days | ND | Bioluminescence | Bioluminescence per relative radiance and mean radiance | ND |
| Chaudhury *et al.* 2012 | 15 mg/kg | Twice weekly for three weeks (on day 14, 18, 21, 25, 28 and 32) | 14 days | 60 days | Euthanized when severe ill or showing weight loss/gain of 20% | na | Survival and body weight change | Physical activity, pain and disease progression |
| Cho *et al.* 2013 | 30 mg/kg of PTX, CYP and GSP | Treatment at day 0, 7, 14 after inoculation time | ES-2: 4 days post inoculation  SKOV-3: 16 days post inoculation | 2 months | Euthanized when reaching moribund conditions | Measuring abdominal radii with portable scale and digital caliper. Whole body bioluminescence. Whole-body microPET/CT and survival | Survival, bioluminescence imaging, abdominal radius, body weight change and PetCT imaging | ND |
| Cho *et al.* 2014 | 30 mg/kg | 1 treatment 7 days post inoculations. The next day imaging treatment | 7 days | ND | ND | Bioluminescence and body weight | Bioluminescence | Body weight, general appearance, mortality |
| Cirstoiu-Hapca *et al.* 2010 | Initial dose: 20 mg/kg (10 mg/kg IP and 10 mg/kg IV) Subsequent doses 10 mg/kg IP or IV | Bioluminescence: day 5: IV+IP; day 8: IP; day 11: IV; day14: IP; day 17: IV; day 20 IP.  Survival rate: day 3: IV+IP; day 6: IP; day 9: IV; day 12: IP; day 15: IV; day 8: IP | Bioluminescence: 5 days post inoculation. Survival rate: 3 days post inoculation | Bioluminescence: 70 days post inoculation. Survival rate: 53 days post inoculation | Only for the survival rate group: euthanized when presented inactivity, hunched position, spiky hair, or 20% weight loss/gain | Bioluminescence and signal imaging | Bioluminescence signal, survival rate and body weight | ND |
| Daoud 1994 | Valinomycin-liposomes: 1 mg/kg; CIS: 3 mg/kg or 1 mg/kg, and a combination of valinomycin liposomes (1 mg/kg) and free CIS (1 mg/kg) | First 24 h after inoculation. Then every 5^th^ day for 2 weeks | 24 h | 150 days after tumor inoculation | Mortality recorded daily | ND | Survival rate | Urea nitrogen, plasma creatinine, alanine aminotransferase, alkaline phosphatase, blood glucose and plasma electrolytes. |
| Gao *et al.* 2005 | 3 mg/kg | Treatment on day 1, 4, 7 and 11 post-inoculation. Treatment on day 1, 26 and 35 post-inoculation for conditions: free DOX, free DOX+US and DOX/micelles+US | 1 day | ND; max survival = 118 days | Sacrificed when tumor reached 15% of body weight | Tumor yield; method ND | Tumor yield and survival rate | Sacrificed when tumor reached 15% of body weight |
| Gharpure *et al.* 2014 | 0.5, 1, 2 and 20 mg/kg | Dose-response study: MTD once in 2 weeks. Other doses 3 times a week.  Therapeutic experiment: siRNA twice a week; PLGA-PRINT docetaxel 3 times weekly | 1 week | ND | All mice sacrificed when a mouse seemed to be moribund. | Bioluminescence, body weight, tumor weight and number of tumor nodules | Bioluminescence, body weight, tumor weight and number of tumor nodules | Monitored for toxic adverse effects. Details not stated |
| Gilmore *et al.* 2012 | 10 mg/kg | Four weeks after established disease, operative debulking consisting of midline laparotomy, oophorectomy, omentectomy, and resection of macroscopic tumor nodules was performed | 4 weeks | 4 weeks | All animals were sacrificed when non-drug controls displayed evidence of disease progression | Tumor mass and bioluminescence | Body weight; tumor mass; %animals with significant recurrence | Weight loss, ascites, intra-abdominal tumor registration |
| Javid *et al.* 2014 | 10 mg/kg | 1x 30 days after inoculation | 30 days | 40 days | ND | Measurement of radius and survival | Tumor volume and survival | ND |
| Jin *et al.* 2007 | 10 mg/kg | 4, 5 and 6 weeks post inoculation | 4 weeks | 6 weeks + 6h post inoculation | ND | Photographs of intestine and mesentery. Images analyzed for tumor number, average tumor diameter, area of photograph occupied by tumors, and total area of the image occupied by intestine/mesentery | Tumor numbers, size and areas | ND |
| Konishi *et al.* 2012 | 45 mg/kg | Treatment at day 4, 8 and 12 post inoculation.  For additional dosing experiment: treatment at day 4, 8, 12 or 8, 12, 16 or 12, 16, 20 (only IHL-305 group) | 4 days | Evaluation at day 18, 26, 31 | ND | Monitoring the volume of ascites and tumor nodules in omentum, diaphragm, mesenterium and pancreas | Survival, ascites incidence and volume, and metastasis incidence | ND |
| Lee *et al.* 2013 | PTX-NS: 25 mg/kg (IP)  PTX: 15 mg/kg (IV) | PTX-ns: day 1 and 14  Free PTX: every 3 days, for a total of 5 times.  Also a combination of the two was applied | 6 weeks | 296 days after inoculation | ND | Whole body RFP imaging. Survived animals were sacrificed at day 296, tumor masses collected and examined by routine histology | RFP imaging, survival and body weight | Body weight |
| Li and Howell 2010 | Free CIS: 10 mg/kg  Hyplat: 5 mg CIS/ kg | 1 IP injection 14 days after inoculation | 14 days | Evaluation at day 45 | When mice lost >20% of their initial body weight due to disease progression or became moribund, they were euthanized | Bioluminescence | Bioluminescence, survival rate and body weight | Body weight |
| Lu *et al.* 2006 | 5 mg/kg | Once weekly for 5 weeks | ND | 5 weeks | ND | Tumor load and ascites volume | Tumor load and ascites volume | ND |
| Lu *et al.* 2007 | 5 mg/kg | Once weekly for 5 weeks | 4 weeks | 5 weeks | ND | Tumor weight and ascites volume | Tumor weight and ascites volume | Body weight |
| Lu *et al.* 2008 | Free PTX: 4, 8 or 40 mg/kg. Priming TPM: 40 mg/kg. Sustaining TPM: 80 mg/kg. Priming/sustaining TPM: 120 mg/kg | Free PTX: 8 doses of 15 mg/kg, 4 doses of 10 mg/kg or a single dose of 40 mg/kg. Priming TPM: single dose of 40 mg/kg). Sustaining TPM: single dose of 80 mg/kg. Priming/sustaining TPM: single dose of 1:2 priming /sustaining TPM 120 mg/kg) | 28 days | 175 days | Treatment related deaths (>15% body weight loss, internal hemorrhage) if <10 days after treatment.  Disease related deaths: from 10 days onwards with presence of tumor nodules | na | Body weight change, Cure%, median survival time and increase in life-span | Weight loss, gastrointestinal toxicity and adhesion |
| Mantia-Smaldone *et al.* 2014 | 50 mg/m^2^ | A total of 3 injections on days 3, 10, and 17 | 3 days | 80 days | Euthanized when a weight of 30 gram was reached. Animals with evidence of distress or toxicity before reaching 30 gram body weight were euthanized and necropsy was performed | na | Survival | ND |
| Mignard *et al.* 2010 | 1, 2.5 or 3 mg/kg | 2.5 mg/kg every 4 days for 3 weeks. 1 and 3 mg/kg every 7 days for 3 weeks.  Taxol every 7 days for 4 weeks | 1 day | 100 days | ND | Body weight and tumor volume | Antitumor activity (optimal treated/control percentage) | Tolerance |
| Paraskar *et al.* 2010 | 1.25 or 3 mg/kg CIS | ND | Once mice developed medium to large tumors | ND | ND | Bioluminescence | Bioluminescence signal and body weight | Body weight, liver and spleen examination and TUNEL assay of vital organs. Nephrotoxicity. |
| Pastorino *et al.* 2008 | 5 mg/kg (max tolerated dose); 3.3 mg/kg; 1.7 mg/kg | Once weekly for 5 weeks | 5 or 7 days | 125 days | Euthanized when body weight and physical status (abdominal dilatation, dehydration and paraplegia) were judged to be in discomfort | na | Survival distribution; survival time | Body weight and physical status |
| Patankar *et al.* 2013 | Free topotecan: 5 mg/kg  Topophore C: 1.25; 2.5 and 5 mg/kg | Every 7 days for 3 weeks | 7 days | 60 days | Daily monitoring of body conditions and weight, tumor growth and morbidities. Euthanized when reaching humane endpoints. Necropsies were performed on sacrificed animals | Survival: overall health used to assess morbidity. Using a scoring systems it was determined if mice should be sacrificed. Day of death was reported as 1 day following termination due to morbidity. | Survival and increase in life-span | Weight loss |
| Patankar *et al.* 2013-1 | ES-2 model: Various single agent doses of free topotecan (5-120 mg/kg) and topophore C (2.5-7.5 mg/kg) compared to Doxil (7.5 mg/kg), various combinations of free topotecan (5-15 mg/kg) or topophore C (0.625-5 mg/kg) with Doxil (7.5 mg/kg)  SKOV-3 model:  free topotecan (15 mg/kg), topophore (2.5 mg/kg), Doxil (75 mg/kg), Docil+free topotecan, Doxil+topophore | Every 7 days for 3 weeks | 7 days | SKOV3 group: 42 days | Overall health parameters were used to assess morbidity. Using a scoring system it was determined whether a mouse should be sacrificed. | ES-2 model: survival: Day of death was reported as 1 day following termination due to morbidity.  Necropsy was performed on sacrificed animals.  SKOV-3 model: Bioluminescence | Survival, increase in life-span and bioluminescence | ND |
| Perkins *et al.* 2000 | BrC16-T: 12.5; 25; 50; 100 mg/kg.  Free PTX: 12.5 and 25 mg/kg. | IP study: treatment on day 20, 22, 24, 26, 28 days after inoculation  IV study: treatment on day 1, 3, 5, 7 and 9 days after inoculation | IP study: 20 days. IV study: 1 day | 300 days | ND | na | Survival | ND |
| Pu *et al.* 2014 | 20 mg/kg | Every 3 days for a total of 15 days | 15 days | 2 mice/group sacrificed 24 h after last treatment | ND | na | Survival | Body weight and physical activity |
| Rapoport *et al.* 2004 | 3 mg/kg | 1 day after tumor inoculation, then repeated on day 30 and 40 | 1 day | ND | ND | na | Survival | ND |
| Sengupta *et al.* 2012 | 3 mg/kg | 3 times over a 6 day period (1 day interval) | Once mice developed medium to large tumors | 1 day following last treatment | ND | Quantification luciferase signal | Bioluminescence | ND |
| Shaikh *et al.* 2013 | 10 µmol/kg | Treatment at day 14, 17 and 23 | 14 days | 60 days post inoculation | Mice were euthanized when weight loss >20%, scruffy coat, hunched appearance and moribund state | Survival. If euthanized, dead was defined as the day following euthanization | Survival | Body weight and general condition |
| Storm *et al.* 1994 | 0.67 mg dox/kg in 25 ml/kg | One injection 3 days after inoculation | 3 days | 21 days after inoculation | ND | Tumor weight | Tumor mass | ND |
| Tang *et al.* 2012 | 2 µg/g | One treatment a day for 5 days at day 2 after inoculation, followed by once a day for 5 days after day 14. | 5 days | At day 30 half of mice were sacrificed for tumor detection. Rest used for survival study (80 days). | ND | Tumor nodules observed macroscopically | Tumor nodules number and survival time | Liver and kidney function: data not shown |
| Tong *et al.* 2014 | 20 mg/kg | Weekly for 4 weeks | 1 week | 3 months | Monitoring for abdominal circumference, body weight and mental state until death or sacrifice. | Tumor nodules were counted from the surface of tissues and organs in the peritoneal cavity and processed for histology and flow cytometry | Tumor nodules and survival | Fur color, abdominal circumference, body weight, and mental state biweekly |
| Ueno *et al.* 1988 | 5 mg/kg | Every 4 days 3 times in total | When the transplant reached 250-300 mm^3^ | 12 days | ND | Tumor weight | Tumor growth reduction rate | ND |
| Vaage *et al.* 1993 | 9 mg/kg | Treatment on day 1, 8 and 15 after inoculation | 1 day | 70 days | Euthanized when showing signs of discomfort or when palpation determined progressive tumor growth. Necropsy was performed | na | Survival | ND |
| Vingerhoeds *et al.* 1996 | 0, 0.67, 2, 6, 18 mg/kg in 0.5 ml total volume | Single injection | 7 days | 3 weeks after inoculation | ND | Peritoneal cavity was rinsed with PBS to collect all free-floating cells. Cells were centrifuged and the pellet weight was determined | Weight of collected tumor cells | ND |
| Werner *et al.* 2011 | 20 µg PTX | 1 injection, 3 weeks after inoculation | 3 weeks | 90 days post treatment or after meeting euthanasia criteria | Euthanized when tumor burden or ascites fluid reached non-specified guidelines | Dissection to identify residual tumors | Survival | ND |
| Winer *et al.* 2010 | Free CIS: 250 µg/kg  CIS in nanoparticles: 150 µg/kg for IP or IV; 75 µg/kg if administrated IP and IV (total 150 µg/kg) | Treatment at day 10, 14, 21 and 28 post inoculation | 10 days | 60 days | Sacrificed after 10 gram weight gain or if appeared moribund. | na | Survival | ND |
| Xiao *et al.* 2009 | Taxol 20 mg/kg  Abraxane 45 mg/kg  PTX-np: 20 or 45 mg/kg | Treatment at day 0, 4, 8, 12 and 16 | 3 weeks | 115 days | Euthanized when moribund | Weekly semi quantitative by bioluminescence | Bioluminescence signal and survival | ND |
| Xu *et al.* 2006 | 10 mg/kg | Treated at 4 and 5 weeks post inoculation | 4 weeks post inoculation | 6 weeks post inoculation or 1-2 weeks after treatment: not clear | ND | Counting tumor nodules along a 1-cm^2^ segment of intestine/mesentery at each of three different site per animal | Tumor nodule number | ND |
| Yang *et al.* 2014 | 20 mg/kg | 1 single dose 4 weeks after inoculation | 4 weeks | > 60 days | Mice with high tumor loads (bioluminescence value >1.5·10^8^ p/s) and/or severe ascites were euthanized | Bioluminescence examined weekly | Tumor size and survival | ND |
| Ye *et al.* 2013 | 5 mg/kg | Once weekly for 5 weeks | 4 weeks post inoculation when tumor was visible in abdominal cavity | 5 weeks after treatment start (1 h after the final dose) | ND | Ascites volume using a syringe and tumors collected on ovaries, peritoneum and omentum were weighed | Tumor weight, ascites volume and side effects | Hematology (red blood cell, hemoglobin, white blood cell and platelets), creatine kinase, aspartate aminotransferase and lactate dehydrogenase, serum cardiac enzyme, Bone marrow smear, heart histopathology and body weight |
| Zeng *et al.* 2013 | 5 mg/kg | Every 5 days | After the development of ascites | ND | Euthanized when expected to become moribund within short time | Measuring abdominal circumference using a soft ruler. Tumor and heart tissue removal for histology | Histological alterations and assessment of apoptosis in tumors after treatment | Histology of heart tissue |
| Zhang *et al.* 2013 | 4 mg/kg AT | 5 times every other day | 4 days | 2 days after last treatment (day 14) | ND | ND | Tumor weight | Blood toxicity analysis in additional study |

**Abbreviations:** AHM = 3-(acryloyloxy)-2-hydroxypropylmethacryamide, APMA = 3-(aminopropyl)methacrylamide, APTES = (3-aminopropyl)Triethoxysilane, AT = Arsenic Trioxide, AOT = dioctylsulfosuccinate, Br-16-PTX = Bromohexadecanoyl-PTX, Brij = PEG-dodecyl ether, CHOL = Cholesterol, CIS = Cisplatin, CPT = carboplatin, CYP = Cyclopamine, DiR = (1,19-dioctadecyltetramethyl indotricarbocyanine iodide), DMPS = Dimyristoylphosphatidylcholine, DOC = Docetaxel, DOTAP = 1,2-Dioleoyl-3-Trimethylammonium-propane, DOX = Doxorubicin, DP = Dicetyl phosphate, DPPC = Dipalmitoylphosphatidylcholine, DPPE-GA = 1,2-Dihexadecanoyl-sn-glycero-3-phosphoethanolamine-N-(glutaryl), DSPC =1,2-Distearoyl-sn-glycero-3-phosphocholine, DSPE = 1,2-distearoyl-sn-glycero-3-phosphatidyl-ethanolamine, EPG = Phosphatidylglycerol, F = Female, FA = Folate, FRa = Folate Receptor a, GSP = Gossypol, HSPC = Hydrogenated soy phosphatidylcholine, ITC = Irinotecan, LHRHa/r = Luteinizing hormone-releasing hormone analogue/receptor, M = Male, MPB = N-[4-(p-maleimidophenyl)butyryl], Na = Not applicable, ND = not described, DCM = dichloromethane NGR = aspargine-glycine-arginine, PAE = Poly(β-aminoester), PBS = phosphate buffered saline, PC = Phosphatidylcholine, PCL = Poly(e-caprolactone), PDEA = Poly[2-(N,N-diethylamino)ethyl methacrylate], PDLA = Poly-D-lactic acid, PDMA = poly[2-(N,N-dimethylamino)ethyl methacrylate], PE = Posphatidylethanolamine, PEG = Poly-ethyleneglycol, PEO-PPO-PEO = Poly(ethylene oxide)-co-poly(propylene oxide)-co-(polyethylene oxide) triblock copolymer (Pluronic P-105 block polymer), PIMA-GA = Poly-isobutylene-maleic acid – glucosamine, PLA = Polylactic acid, PLGA = Poly(lactic-co-glycolic acid), PS = Phosphatidylserine, PSA = Poly(sebacic acid), PTX = Paclitaxel SPIO = Superparamagnetic iron oxide, TOP = Tropophore C, uPA = urokinase Plasminogen activator.

## References

[1] I. Alagkiozidis, A. Facciabene, C. Carpenito, F. Benencia, Z. Jonak, S. Adams, R.G. Carroll, P.A. Gimotty, R. Hammond, G.A. Danet-Desnoyers, C.H. June, D.J. Powell, Jr., G. Coukos, Increased immunogenicity of surviving tumor cells enables cooperation between liposomal doxorubicin and IL-18, Journal of translational medicine, 7 (2009) 104.

[2] S. Ali, I. Ahmad, A. Peters, G. Masters, S. Minchey, A. Janoff, E. Mayhew, Hydrolyzable hydrophobic taxanes: synthesis and anti-cancer activities, Anti-cancer drugs, 12 (2001) 117-128.

[3] D.D. Ateh, V.H. Leinster, S.R. Lambert, A. Shah, A. Khan, H.J. Walklin, J.V. Johnstone, N.I. Ibrahim, M.M. Kadam, Z. Malik, M. Girones, G.J. Veldhuis, G. Warnes, S. Marino, I.A. McNeish, J.E. Martin, The intracellular uptake of CD95 modified paclitaxel-loaded poly(lactic-co-glycolic acid) microparticles, Biomaterials, 32 (2011) 8538-8547.

[4] A. Chaudhury, S. Das, R.M. Bunte, G.N. Chiu, Potent therapeutic activity of folate receptor-targeted liposomal carboplatin in the localized treatment of intraperitoneally grown human ovarian tumor xenograft, International journal of nanomedicine, 7 (2012) 739-751.

[5] H. Cho, T.C. Lai, G.S. Kwon, Poly(ethylene glycol)-block-poly(epsilon-caprolactone) micelles for combination drug delivery: evaluation of paclitaxel, cyclopamine and gossypol in intraperitoneal xenograft models of ovarian cancer, Journal of controlled release : official journal of the Controlled Release Society, 166 (2013) 1-9.

[6] H. Cho, C.S. Cho, G.L. Indig, A. Lavasanifar, M.R. Vakili, G.S. Kwon, Polymeric micelles for apoptosis-targeted optical imaging of cancer and intraoperative surgical guidance, PloS one, 9 (2014) e89968.

[7] A. Cirstoiu-Hapca, F. Buchegger, N. Lange, L. Bossy, R. Gurny, F. Delie, Benefit of anti-HER2-coated paclitaxel-loaded immuno-nanoparticles in the treatment of disseminated ovarian cancer: Therapeutic efficacy and biodistribution in mice, Journal of controlled release : official journal of the Controlled Release Society, 144 (2010) 324-331.

[8] S.S. Daoud, Combination chemotherapy of human ovarian xenografts with intraperitoneal liposome-incorporated valinomycin and cis-diamminedichloroplatinum(II), Cancer chemotherapy and pharmacology, 33 (1994) 307-312.

[9] Z.G. Gao, H.D. Fain, N. Rapoport, Controlled and targeted tumor chemotherapy by micellar-encapsulated drug and ultrasound, Journal of controlled release : official journal of the Controlled Release Society, 102 (2005) 203-222.

[10] K.M. Gharpure, K.S. Chu, C.J. Bowerman, T. Miyake, S. Pradeep, S.L. Mangala, H.D. Han, R. Rupaimoole, G.N. Armaiz-Pena, T.B. Rahhal, S.Y. Wu, J.C. Luft, M.E. Napier, G. Lopez-Berestein, J.M. DeSimone, A.K. Sood, Metronomic docetaxel in PRINT nanoparticles and EZH2 silencing have synergistic antitumor effect in ovarian cancer, Molecular cancer therapeutics, 13 (2014) 1750-1757.

[11] D. Gilmore, M. Schulz, R. Liu, K.A. Zubris, R.F. Padera, P.J. Catalano, M.W. Grinstaff, Y.L. Colson, Cytoreductive surgery and intraoperative administration of paclitaxel-loaded expansile nanoparticles delay tumor recurrence in ovarian carcinoma, Annals of surgical oncology, 20 (2013) 1684-1693.

[12] A. Javid, S. Ahmadian, A.A. Saboury, S.M. Kalantar, S. Rezaei-Zarchi, S. Shahzad, Biocompatible APTES-PEG modified magnetite nanoparticles: effective carriers of antineoplastic agents to ovarian cancer, Applied biochemistry and biotechnology, 173 (2014) 36-54.

[13] W. Jin, P. Xu, Y. Zhan, Y. Shen, E.A. Van Kirk, B. Alexander, W.J. Murdoch, L. Liu, D.D. Isaak, Degradable cisplatin-releasing core-shell nanogels from zwitterionic poly(beta -aminoester)-graft-PEG for cancer chemotherapy, Drug delivery, 14 (2007) 279-286.

[14] H. Konishi, A. Takagi, A. Kurita, N. Kaneda, T. Matsuzaki, PEGylated liposome IHL-305 markedly improved the survival of ovarian cancer peritoneal metastasis in mouse, BMC cancer, 12 (2012) 462.

[15] S.E. Lee, S.F. Bairstow, J.O. Werling, M.V. Chaubal, L. Lin, M.A. Murphy, J.P. DiOrio, J. Gass, B. Rabinow, X. Wang, Y. Zhang, Z. Yang, R.M. Hoffman, Paclitaxel nanosuspensions for targeted chemotherapy - nanosuspension preparation, characterization, and use, Pharmaceutical development and technology, 19 (2014) 438-453.

[16] S.D. Li, S.B. Howell, CD44-targeted microparticles for delivery of cisplatin to peritoneal metastases, Molecular pharmaceutics, 7 (2010) 280-290.

[17] H.X. Lu, C.J. Xu, B. Li, Y. Kang, Q. Huang, L.M. Li, Q.H. Chen, [The inhibitory effect of paclitaxel nanoparticles on ovarian cancer xenografts and lymphatic targeting], Beijing da xue xue bao. Yi xue ban = Journal of Peking University. Health sciences, 38 (2006) 483-486.

[18] H. Lu, B. Li, Y. Kang, W. Jiang, Q. Huang, Q. Chen, L. Li, C. Xu, Paclitaxel nanoparticle inhibits growth of ovarian cancer xenografts and enhances lymphatic targeting, Cancer chemotherapy and pharmacology, 59 (2007) 175-181.

[19] Z. Lu, M. Tsai, D. Lu, J. Wang, M.G. Wientjes, J.L. Au, Tumor-penetrating microparticles for intraperitoneal therapy of ovarian cancer, The Journal of pharmacology and experimental therapeutics, 327 (2008) 673-682.

[20] G. Mantia-Smaldone, L. Ronner, A. Blair, V. Gamerman, C. Morse, S. Orsulic, S. Rubin, P. Gimotty, S. Adams, The immunomodulatory effects of pegylated liposomal doxorubicin are amplified in BRCA1--deficient ovarian tumors and can be exploited to improve treatment response in a mouse model, Gynecologic oncology, 133 (2014) 584-590.

[21] C. Mignard, V. Bari, L. Bichat, L. Cicurel, G. Vuagniaux, M. Barbier, PK and antitumor activity of DEBIO 0507, a new platinum derivative, in preclinical tumor models, in: 101st Annual Meeting of the American Association for Cancer Research, American Association for Cancer Research Inc., AACR 2010 Washington, DC United States, 2010.

[22] A.S. Paraskar, S. Soni, K.T. Chin, P. Chaudhuri, K.W. Muto, J. Berkowitz, M.W. Handlogten, N.J. Alves, B. Bilgicer, D.M. Dinulescu, R.A. Mashelkar, S. Sengupta, Harnessing structure-activity relationship to engineer a cisplatin nanoparticle for enhanced antitumor efficacy, Proceedings of the National Academy of Sciences of the United States of America, 107 (2010) 12435-12440.

[23] F. Pastorino, D. Di Paolo, F. Piccardi, B. Nico, D. Ribatti, A. Daga, G. Baio, C.E. Neumaier, C. Brignole, M. Loi, D. Marimpietri, G. Pagnan, M. Cilli, E.A. Lepekhin, S.V. Garde, R. Longhi, A. Corti, T.M. Allen, J.J. Wu, M. Ponzoni, Enhanced antitumor efficacy of clinical-grade vasculature-targeted liposomal doxorubicin, Clinical cancer research : an official journal of the American Association for Cancer Research, 14 (2008) 7320-7329.

[24] N.A. Patankar, D. Waterhouse, D. Strutt, M. Anantha, M.B. Bally, Topophore C: a liposomal nanoparticle formulation of topotecan for treatment of ovarian cancer, Investigational new drugs, 31 (2013) 46-58.

[25] N.A. Patankar, J. Pritchard, M. van Grinsven, M. Osooly, M.B. Bally, Topotecan and doxorubicin combination to treat recurrent ovarian cancer: the influence of drug exposure time and delivery systems to achieve optimum therapeutic activity, Clinical cancer research : an official journal of the American Association for Cancer Research, 19 (2013) 865-877.

[26] W.R. Perkins, I. Ahmad, X. Li, D.J. Hirsh, G.R. Masters, C.J. Fecko, J. Lee, S. Ali, J. Nguyen, J. Schupsky, C. Herbert, A.S. Janoff, E. Mayhew, Novel therapeutic nano-particles (lipocores): trapping poorly water soluble compounds, International journal of pharmaceutics, 200 (2000) 27-39.

[27] C. Pu, S. Chang, J. Sun, S. Zhu, H. Liu, Y. Zhu, Z. Wang, R.X. Xu, Ultrasound-mediated destruction of LHRHa-targeted and paclitaxel-loaded lipid microbubbles for the treatment of intraperitoneal ovarian cancer xenografts, Molecular pharmaceutics, 11 (2014) 49-58.

[28] N.Y. Rapoport, D.A. Christensen, H.D. Fain, L. Barrows, Z. Gao, Ultrasound-triggered drug targeting of tumors in vitro and in vivo, Ultrasonics, 42 (2004) 943-950.

[29] P. Sengupta, S. Basu, S. Soni, A. Pandey, B. Roy, M.S. Oh, K.T. Chin, A.S. Paraskar, S. Sarangi, Y. Connor, V.S. Sabbisetti, J. Kopparam, A. Kulkarni, K. Muto, C. Amarasiriwardena, I. Jayawardene, N. Lupoli, D.M. Dinulescu, J.V. Bonventre, R.A. Mashelkar, S. Sengupta, Cholesterol-tethered platinum II-based supramolecular nanoparticle increases antitumor efficacy and reduces nephrotoxicity, Proceedings of the National Academy of Sciences of the United States of America, 109 (2012) 11294-11299.

[30] I.M. Shaikh, K.B. Tan, A. Chaudhury, Y. Liu, B.J. Tan, B.M. Tan, G.N. Chiu, Liposome co-encapsulation of synergistic combination of irinotecan and doxorubicin for the treatment of intraperitoneally grown ovarian tumor xenograft, Journal of controlled release : official journal of the Controlled Release Society, 172 (2013) 852-861.

[31] G. Storm, U.K. Nassander, M.H. Vingerhoeds, P.A. Steerenberg, D.J.A. Crommelin, Antibody-targeted liposomes to deliver doxorubicin to ovarian cancer cells, Journal of liposome research, 4 (1994) 641-666.

[32] K. Tang, Y. Zhang, H. Zhang, P. Xu, J. Liu, J. Ma, M. Lv, D. Li, F. Katirai, G.X. Shen, G. Zhang, Z.H. Feng, D. Ye, B. Huang, Delivery of chemotherapeutic drugs in tumour cell-derived microparticles, Nature communications, 3 (2012) 1282.

[33] L. Tong, W. Chen, J. Wu, H. Li, Folic acid-coupled nano-paclitaxel liposome reverses drug resistance in SKOV3/TAX ovarian cancer cells, Anti-cancer drugs, 25 (2014) 244-254.

[34] N. Ueno, Experimental studies on the chemotherapy of gynecological neoplasm by means of adriamycin entrapped in liposomes, Journal of the Aichi Medical University Association, 16 (1988) 63-82.

[35] J. Vaage, D. Donovan, E. Mayhew, R. Abra, A. Huang, Therapy of human ovarian carcinoma xenografts using doxorubicin encapsulated in sterically stabilized liposomes, Cancer, 72 (1993) 3671-3675.

[36] M.H. Vingerhoeds, P.A. Steerenberg, J.J. Hendriks, L.C. Dekker, Q.G. Van Hoesel, D.J. Crommelin, G. Storm, Immunoliposome-mediated targeting of doxorubicin to human ovarian carcinoma in vitro and in vivo, British journal of cancer, 74 (1996) 1023-1029.

[37] M.E. Werner, S. Karve, R. Sukumar, N.D. Cummings, J.A. Copp, R.C. Chen, T. Zhang, A.Z. Wang, Folate-targeted nanoparticle delivery of chemo- and radiotherapeutics for the treatment of ovarian cancer peritoneal metastasis, Biomaterials, 32 (2011) 8548-8554.

[38] I. Winer, S. Wang, Y.E. Lee, W. Fan, Y. Gong, D. Burgos-Ojeda, G. Spahlinger, R. Kopelman, R.J. Buckanovich, F3-targeted cisplatin-hydrogel nanoparticles as an effective therapeutic that targets both murine and human ovarian tumor endothelial cells in vivo, Cancer research, 70 (2010) 8674-8683.

[39] K. Xiao, J. Luo, W.L. Fowler, Y. Li, J.S. Lee, L. Xing, R.H. Cheng, L. Wang, K.S. Lam, A self-assembling nanoparticle for paclitaxel delivery in ovarian cancer, Biomaterials, 30 (2009) 6006-6016.

[40] P. Xu, E.A. Van Kirk, W.J. Murdoch, Y. Zhan, D.D. Isaak, M. Radosz, Y. Shen, Anticancer efficacies of cisplatin-releasing pH-responsive nanoparticles, Biomacromolecules, 7 (2006) 829-835.

[41] M. Yang, T. Yu, J. Wood, Y.Y. Wang, B.C. Tang, Q. Zeng, B.W. Simons, J. Fu, C.M. Chuang, S.K. Lai, T.C. Wu, C.F. Hung, J. Hanes, Intraperitoneal delivery of paclitaxel by poly(ether-anhydride) microspheres effectively suppresses tumor growth in a murine metastatic ovarian cancer model, Drug delivery and translational research, 4 (2014) 203-209.

[42] L. Ye, J. He, Z. Hu, Q. Dong, H. Wang, F. Fu, J. Tian, Antitumor effect and toxicity of Lipusu in rat ovarian cancer xenografts, Food and chemical toxicology : an international journal published for the British Industrial Biological Research Association, 52 (2013) 200-206.

[43] Q. Zeng, H. Wen, Q. Wen, X. Chen, Y. Wang, W. Xuan, J. Liang, S. Wan, Cucumber mosaic virus as drug delivery vehicle for doxorubicin, Biomaterials, 34 (2013) 4632-4642.

[44] Y. Zhang, H.A. Kenny, E.P. Swindell, A.K. Mitra, P.L. Hankins, R.W. Ahn, K. Gwin, A.P. Mazar, T.V. O'Halloran, E. Lengyel, Urokinase plasminogen activator system-targeted delivery of nanobins as a novel ovarian cancer therapy, Molecular cancer therapeutics, 12 (2013) 2628-2639.
